# *tert*-Butyl 6-acetamido-3,4-dihydro-2*H*-1,4-benzoxazine-4-carboxyl­ate

**DOI:** 10.1107/S1600536810036937

**Published:** 2010-09-25

**Authors:** Xiao-Bo Gu

**Affiliations:** a Key Laboratory of Nuclear Medicine, Ministry of Health, Jiangsu Key Laboratory of Molecular Nuclear Medicine, Jiangsu Institute of Nuclear Medicine, Wuxi 214063, People’s Republic of China

## Abstract

The title mol­ecule, C_15_H_20_N_2_O_4_, contains a benzene ring fused to an oxazine ring and one *tert*-but­oxy­carbonyl group bound to the N atom. An intra­molecular C—H⋯O inter­action occurs. In the crystal, mol­ecules are linked through inter­molecular N—H⋯O and C—H⋯O hydrogen bonds.

## Related literature

For the pharmacological properties of phenyl­morpholine derivatives, see: Bourlot *et al.* (1998 ▶); Albanese *et al.* (2003 ▶); La *et al.* (2008 ▶). For structures, see: Chen *et al.* (2003 ▶); Olmstead *et al.* (2003 ▶); Vergeer *et al.* (1999 ▶).
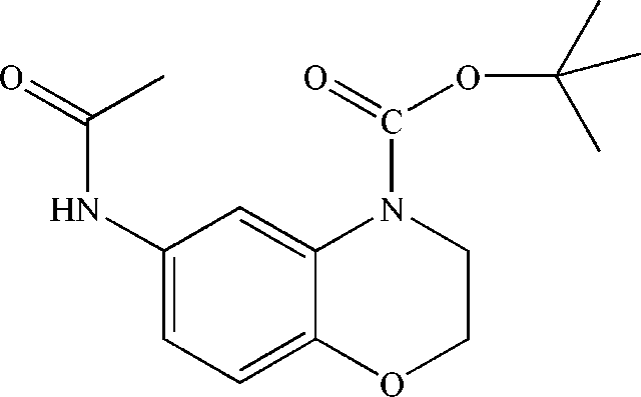


## Experimental

### 

#### Crystal data

C_15_H_20_N_2_O_4_
*M* *_r_* = 292.33
Orthorhombic, 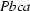

*a* = 9.675 (2) Å
*b* = 13.137 (3) Å
*c* = 23.128 (5) Å
*V* = 2939.7 (12) Å^3^
*Z* = 8
Mo *K*α radiation
μ = 0.10 mm^−1^
*T* = 103 K
0.43 × 0.40 × 0.18 mm

#### Data collection

Rigaku SPIDER diffractometer
20907 measured reflections
3367 independent reflections
3005 reflections with *I* > 2σ(*I*)
*R*_int_ = 0.039

#### Refinement

*R*[*F* ^2^ > 2σ(*F* ^2^)] = 0.048
*wR*(*F* ^2^) = 0.114
*S* = 1.00
3367 reflections
198 parameters
H atoms treated by a mixture of independent and constrained refinement
Δρ_max_ = 0.29 e Å^−3^
Δρ_min_ = −0.28 e Å^−3^

### 

Data collection: *RAPID-AUTO* (Rigaku, 2004 ▶); cell refinement: *RAPID-AUTO*; data reduction: *RAPID-AUTO*; program(s) used to solve structure: *SHELXS97* (Sheldrick, 2008 ▶); program(s) used to refine structure: *SHELXL97* (Sheldrick, 2008 ▶); molecular graphics: *SHELXTL* (Sheldrick, 2008 ▶); software used to prepare material for publication: *SHELXTL*.

## Supplementary Material

Crystal structure: contains datablocks I, global. DOI: 10.1107/S1600536810036937/jh2200sup1.cif

Structure factors: contains datablocks I. DOI: 10.1107/S1600536810036937/jh2200Isup2.hkl

Additional supplementary materials:  crystallographic information; 3D view; checkCIF report

## Figures and Tables

**Table 1:** Hydrogen-bond geometry (Å, °)

*D*—H⋯*A*	*D*—H	H⋯*A*	*D*⋯*A*	*D*—H⋯*A*
N2—H2*N*⋯O4^i^	0.879 (19)	2.051 (19)	2.9251 (17)	173.1 (16)
C5—H5⋯O4	0.95	2.27	2.8771 (19)	121
C11—H11*B*⋯O3^ii^	0.98	2.49	3.456 (2)	168

Symmetry codes: (i) 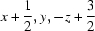; (ii) 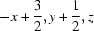.

**Fig. 1.**
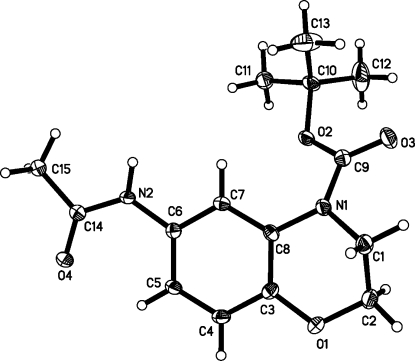
A view of the title compound with the atomic numbering scheme. Displacement ellipsoids were drawn at the 50% probability level.

## supplementary crystallographic information

### Comment

The stucture of the title compound, (I), is an important phenylmorpholine product. Phenylmorpholine compounds are used as α2 C adrenergic receptor agonists. Numerous phenylmorpholine derivatives possess various pharmacological properties (Bourlot, *et al.*, 1998; Albanese, *et al.*, 2003; La *et al.*, 2008).

We report here the crystal structure of the title compound. (Fig. 1). The title molecule of (I) contains a benzene ring fused to an oxazine ring and one *tert*-butoxycarbonyl bound to the N atom. The N1—C8 bond distance is 1.4208 (18) Å and agrees with literature values (Vergeer, *et al.*, 1999; Chen, *et al.*, 2003; Olmstead, *et al.*, 2003). The conformation of the six-membered heterocyclic ring shows that C1 and C2 are out of the plane of the remaining four atoms by 0.5950 (16) and -0.0818 (16) Å, respectively.

The molecules are linked through hydrogen-bonding interactions of types N—H···O and C—H···O (Table 1).

### Experimental

The title compound was crystallized from a mixed solvent composed of dichloromethane and hexane (1:1); colorlessblock-shaped crystals were obtained after several days.

### Refinement

Positional parameters of all the H atomsbonded to C atoms were calculated geometrically and were allowed to ride on the C atoms to which they were bonded, with C—H distances of 0.95Å (CH), 0.98Å (CH_3_) or 0.99Å (CH_2_), and with *U*_iso_(H) =1.2*U*_eq_of the parent atoms. The H-atoms bonded to N atoms was taken from a difference map and was allowed to refine freely.

### Crystal data

**Table:**

C_15_H_20_N_2_O_4_	*F*(000) = 1248
*M**_r_* = 292.33	*D*_x_ = 1.321 Mg m^−^^3^
Orthorhombic, *P**b**c**a*	Mo *K*α radiation, λ = 0.71073 Å
Hall symbol: -P 2ac 2ab	Cell parameters from 8192 reflections
*a* = 9.675 (2) Å	θ = 3.1–27.5°
*b* = 13.137 (3) Å	µ = 0.10 mm^−^^1^
*c* = 23.128 (5) Å	*T* = 103 K
*V* = 2939.7 (12) Å^3^	Chunk, colorless
*Z* = 8	0.43 × 0.40 × 0.18 mm

### Data collection

**Table:**

Rigaku SPIDER diffractometer	3005 reflections with *I* > 2σ(*I*)
Radiation source: Rotating Anode	*R*_int_ = 0.039
graphite	θ_max_ = 27.5°, θ_min_ = 3.1°
ω scans	*h* = −12→12
20907 measured reflections	*k* = −16→17
3367 independent reflections	*l* = −29→29

### Refinement

**Table:**

Refinement on *F*^2^	Primary atom site location: structure-invariant direct methods
Least-squares matrix: full	Secondary atom site location: difference Fourier map
*R*[*F*^2^ > 2σ(*F*^2^)] = 0.048	Hydrogen site location: inferred from neighbouring sites
*wR*(*F*^2^) = 0.114	H atoms treated by a mixture of independent and constrained refinement
*S* = 1.00	*w* = 1/[σ^2^(*F*_o_^2^) + (0.0536*P*)^2^ + 1.960*P*] where *P* = (*F*_o_^2^ + 2*F*_c_^2^)/3
3367 reflections	(Δ/σ)_max_ < 0.001
198 parameters	Δρ_max_ = 0.29 e Å^−^^3^
0 restraints	Δρ_min_ = −0.28 e Å^−^^3^

### Special details

**Table:**

Geometry. All e.s.d.'s (except the e.s.d. in the dihedral angle between two l.s. planes) are estimated using the full covariance matrix. The cell e.s.d.'s are taken into account individually in the estimation of e.s.d.'s in distances, angles and torsion angles; correlations between e.s.d.'s in cell parameters are only used when they are defined by crystal symmetry. An approximate (isotropic) treatment of cell e.s.d.'s is used for estimating e.s.d.'s involving l.s. planes.
Refinement. Refinement of *F*^2^ against ALL reflections. The weighted *R*-factor *wR* and goodness of fit *S* are based on *F*^2^, conventional *R*-factors *R* are based on *F*, with *F* set to zero for negative *F*^2^. The threshold expression of *F*^2^ > σ(*F*^2^) is used only for calculating *R*-factors(gt) *etc*. and is not relevant to the choice of reflections for refinement. *R*-factors based on *F*^2^ are statistically about twice as large as those based on *F*, and *R*- factors based on ALL data will be even larger.

### Fractional atomic coordinates and isotropic or equivalent isotropic displacement parameters (Å^2^)

**Table:**

	*x*	*y*	*z*	*U*_iso_*/*U*_eq_	
O1	0.16030 (11)	0.52261 (8)	0.61658 (5)	0.0221 (3)	
O2	0.53747 (10)	0.73649 (7)	0.57927 (4)	0.0173 (2)	
O3	0.62739 (12)	0.58152 (8)	0.55786 (5)	0.0239 (3)	
O4	0.08299 (11)	0.93078 (8)	0.77211 (5)	0.0222 (3)	
N1	0.43426 (13)	0.59636 (9)	0.61318 (5)	0.0164 (3)	
N2	0.28810 (12)	0.88225 (9)	0.73211 (5)	0.0146 (3)	
C1	0.40682 (16)	0.48702 (11)	0.61007 (7)	0.0201 (3)	
H1A	0.4110	0.4569	0.6493	0.024*	
H1B	0.4775	0.4534	0.5857	0.024*	
C2	0.26528 (17)	0.47066 (11)	0.58444 (7)	0.0212 (3)	
H2A	0.2647	0.4954	0.5440	0.025*	
H2B	0.2445	0.3969	0.5839	0.025*	
C3	0.19649 (15)	0.61199 (11)	0.64273 (6)	0.0162 (3)	
C4	0.09200 (15)	0.66258 (11)	0.67188 (6)	0.0178 (3)	
H4	0.0014	0.6348	0.6718	0.021*	
C5	0.11645 (15)	0.75263 (11)	0.70112 (6)	0.0166 (3)	
H5	0.0431	0.7872	0.7200	0.020*	
C6	0.25037 (15)	0.79196 (10)	0.70245 (6)	0.0140 (3)	
C7	0.35627 (14)	0.74149 (10)	0.67329 (6)	0.0145 (3)	
H7	0.4475	0.7681	0.6745	0.017*	
C8	0.33000 (15)	0.65256 (11)	0.64237 (6)	0.0145 (3)	
C9	0.54187 (15)	0.63514 (11)	0.58119 (6)	0.0159 (3)	
C10	0.65864 (16)	0.79588 (12)	0.56032 (7)	0.0200 (3)	
C11	0.60965 (17)	0.90434 (11)	0.56794 (7)	0.0237 (3)	
H11A	0.5321	0.9174	0.5417	0.028*	
H11B	0.6855	0.9512	0.5591	0.028*	
H11C	0.5796	0.9146	0.6080	0.028*	
C12	0.6938 (3)	0.77433 (14)	0.49806 (9)	0.0433 (5)	
H12A	0.7274	0.7043	0.4944	0.052*	
H12B	0.7658	0.8216	0.4851	0.052*	
H12C	0.6111	0.7831	0.4741	0.052*	
C13	0.7775 (2)	0.77283 (15)	0.60145 (11)	0.0425 (5)	
H13A	0.7467	0.7825	0.6414	0.051*	
H13B	0.8548	0.8189	0.5933	0.051*	
H13C	0.8077	0.7022	0.5961	0.051*	
C14	0.20747 (14)	0.94428 (11)	0.76449 (6)	0.0155 (3)	
C15	0.28116 (15)	1.03281 (11)	0.79194 (6)	0.0188 (3)	
H15A	0.2406	1.0966	0.7780	0.023*	
H15B	0.3793	1.0306	0.7816	0.023*	
H15C	0.2715	1.0289	0.8341	0.023*	
H2N	0.375 (2)	0.9008 (13)	0.7287 (8)	0.022 (5)*	

### Atomic displacement parameters (Å^2^)

**Table:**

	*U*^11^	*U*^22^	*U*^33^	*U*^12^	*U*^13^	*U*^23^
O1	0.0204 (6)	0.0195 (5)	0.0263 (6)	−0.0049 (4)	0.0015 (4)	−0.0083 (4)
O2	0.0147 (5)	0.0148 (5)	0.0224 (5)	−0.0012 (4)	0.0056 (4)	0.0001 (4)
O3	0.0232 (6)	0.0193 (5)	0.0292 (6)	0.0037 (5)	0.0103 (5)	−0.0013 (5)
O4	0.0124 (5)	0.0268 (6)	0.0275 (6)	−0.0001 (4)	0.0030 (4)	−0.0073 (5)
N1	0.0167 (6)	0.0136 (6)	0.0189 (6)	0.0006 (5)	0.0039 (5)	−0.0008 (5)
N2	0.0107 (6)	0.0153 (6)	0.0179 (6)	−0.0017 (5)	0.0012 (4)	−0.0010 (5)
C1	0.0240 (8)	0.0134 (7)	0.0230 (8)	0.0007 (6)	0.0048 (6)	0.0001 (6)
C2	0.0268 (8)	0.0163 (7)	0.0205 (7)	−0.0020 (6)	0.0038 (6)	−0.0035 (6)
C3	0.0188 (7)	0.0153 (7)	0.0145 (6)	−0.0021 (6)	−0.0023 (5)	0.0000 (5)
C4	0.0128 (7)	0.0205 (7)	0.0202 (7)	−0.0035 (6)	−0.0006 (5)	0.0001 (6)
C5	0.0120 (6)	0.0187 (7)	0.0191 (7)	−0.0006 (5)	0.0022 (5)	−0.0001 (5)
C6	0.0151 (7)	0.0150 (6)	0.0119 (6)	−0.0003 (5)	−0.0001 (5)	0.0016 (5)
C7	0.0125 (7)	0.0164 (7)	0.0146 (6)	−0.0016 (5)	0.0004 (5)	0.0020 (5)
C8	0.0144 (7)	0.0159 (7)	0.0130 (6)	0.0019 (5)	0.0016 (5)	0.0020 (5)
C9	0.0156 (7)	0.0167 (7)	0.0153 (6)	0.0014 (6)	−0.0011 (5)	0.0005 (5)
C10	0.0166 (7)	0.0195 (7)	0.0237 (8)	−0.0036 (6)	0.0076 (6)	0.0011 (6)
C11	0.0250 (8)	0.0182 (7)	0.0280 (8)	−0.0039 (6)	0.0068 (6)	−0.0011 (6)
C12	0.0707 (15)	0.0260 (9)	0.0330 (10)	−0.0041 (10)	0.0303 (10)	−0.0012 (8)
C13	0.0241 (10)	0.0336 (10)	0.0697 (15)	−0.0061 (8)	−0.0121 (9)	0.0070 (10)
C14	0.0146 (7)	0.0170 (7)	0.0148 (7)	0.0012 (5)	−0.0007 (5)	0.0020 (5)
C15	0.0169 (7)	0.0198 (7)	0.0197 (7)	−0.0007 (6)	0.0016 (5)	−0.0034 (6)

### Geometric parameters (Å, °)

**Table:**

O1—C3	1.3664 (17)	C5—C6	1.395 (2)
O1—C2	1.4317 (18)	C5—H5	0.9500
O2—C9	1.3328 (18)	C6—C7	1.394 (2)
O2—C10	1.4749 (17)	C7—C8	1.393 (2)
O3—C9	1.2132 (18)	C7—H7	0.9500
O4—C14	1.2301 (18)	C10—C12	1.506 (2)
N1—C9	1.3752 (19)	C10—C11	1.512 (2)
N1—C8	1.4208 (18)	C10—C13	1.523 (2)
N1—C1	1.4624 (19)	C11—H11A	0.9800
N2—C14	1.3540 (18)	C11—H11B	0.9800
N2—C6	1.4180 (18)	C11—H11C	0.9800
N2—H2N	0.88 (2)	C12—H12A	0.9800
C1—C2	1.508 (2)	C12—H12B	0.9800
C1—H1A	0.9900	C12—H12C	0.9800
C1—H1B	0.9900	C13—H13A	0.9800
C2—H2A	0.9900	C13—H13B	0.9800
C2—H2B	0.9900	C13—H13C	0.9800
C3—C4	1.385 (2)	C14—C15	1.505 (2)
C3—C8	1.398 (2)	C15—H15A	0.9800
C4—C5	1.383 (2)	C15—H15B	0.9800
C4—H4	0.9500	C15—H15C	0.9800
			
C3—O1—C2	117.24 (12)	C3—C8—N1	117.45 (13)
C9—O2—C10	120.86 (11)	O3—C9—O2	125.95 (14)
C9—N1—C8	126.92 (12)	O3—C9—N1	122.72 (13)
C9—N1—C1	118.35 (12)	O2—C9—N1	111.33 (12)
C8—N1—C1	113.88 (12)	O2—C10—C12	111.35 (14)
C14—N2—C6	128.51 (12)	O2—C10—C11	102.40 (12)
C14—N2—H2N	115.7 (12)	C12—C10—C11	111.06 (14)
C6—N2—H2N	115.8 (12)	O2—C10—C13	108.04 (13)
N1—C1—C2	108.92 (12)	C12—C10—C13	112.90 (17)
N1—C1—H1A	109.9	C11—C10—C13	110.57 (14)
C2—C1—H1A	109.9	C10—C11—H11A	109.5
N1—C1—H1B	109.9	C10—C11—H11B	109.5
C2—C1—H1B	109.9	H11A—C11—H11B	109.5
H1A—C1—H1B	108.3	C10—C11—H11C	109.5
O1—C2—C1	111.86 (12)	H11A—C11—H11C	109.5
O1—C2—H2A	109.2	H11B—C11—H11C	109.5
C1—C2—H2A	109.2	C10—C12—H12A	109.5
O1—C2—H2B	109.2	C10—C12—H12B	109.5
C1—C2—H2B	109.2	H12A—C12—H12B	109.5
H2A—C2—H2B	107.9	C10—C12—H12C	109.5
O1—C3—C4	116.16 (13)	H12A—C12—H12C	109.5
O1—C3—C8	124.19 (13)	H12B—C12—H12C	109.5
C4—C3—C8	119.64 (13)	C10—C13—H13A	109.5
C5—C4—C3	121.58 (13)	C10—C13—H13B	109.5
C5—C4—H4	119.2	H13A—C13—H13B	109.5
C3—C4—H4	119.2	C10—C13—H13C	109.5
C4—C5—C6	119.10 (13)	H13A—C13—H13C	109.5
C4—C5—H5	120.4	H13B—C13—H13C	109.5
C6—C5—H5	120.4	O4—C14—N2	123.82 (13)
C7—C6—C5	119.71 (13)	O4—C14—C15	121.00 (13)
C7—C6—N2	116.27 (13)	N2—C14—C15	115.18 (12)
C5—C6—N2	124.02 (13)	C14—C15—H15A	109.5
C8—C7—C6	120.86 (13)	C14—C15—H15B	109.5
C8—C7—H7	119.6	H15A—C15—H15B	109.5
C6—C7—H7	119.6	C14—C15—H15C	109.5
C7—C8—C3	119.04 (13)	H15A—C15—H15C	109.5
C7—C8—N1	123.38 (13)	H15B—C15—H15C	109.5
			
C9—N1—C1—C2	−116.78 (14)	C4—C3—C8—C7	2.4 (2)
C8—N1—C1—C2	53.44 (16)	O1—C3—C8—N1	−0.5 (2)
C3—O1—C2—C1	32.01 (17)	C4—C3—C8—N1	178.35 (12)
N1—C1—C2—O1	−56.42 (16)	C9—N1—C8—C7	−41.1 (2)
C2—O1—C3—C4	177.85 (13)	C1—N1—C8—C7	149.70 (13)
C2—O1—C3—C8	−3.3 (2)	C9—N1—C8—C3	143.12 (14)
O1—C3—C4—C5	178.58 (13)	C1—N1—C8—C3	−26.10 (18)
C8—C3—C4—C5	−0.3 (2)	C10—O2—C9—O3	−15.9 (2)
C3—C4—C5—C6	−1.6 (2)	C10—O2—C9—N1	164.66 (12)
C4—C5—C6—C7	1.5 (2)	C8—N1—C9—O3	179.49 (14)
C4—C5—C6—N2	−178.52 (13)	C1—N1—C9—O3	−11.7 (2)
C14—N2—C6—C7	−178.52 (13)	C8—N1—C9—O2	−1.1 (2)
C14—N2—C6—C5	1.5 (2)	C1—N1—C9—O2	167.70 (12)
C5—C6—C7—C8	0.5 (2)	C9—O2—C10—C12	64.04 (18)
N2—C6—C7—C8	−179.43 (12)	C9—O2—C10—C11	−177.21 (13)
C6—C7—C8—C3	−2.5 (2)	C9—O2—C10—C13	−60.47 (18)
C6—C7—C8—N1	−178.20 (12)	C6—N2—C14—O4	−1.4 (2)
O1—C3—C8—C7	−176.45 (13)	C6—N2—C14—C15	178.02 (13)

### Hydrogen-bond geometry (Å, °)

**Table:**

*D*—H···*A*	*D*—H	H···*A*	*D*···*A*	*D*—H···*A*
N2—H2N···O4^i^	0.879 (19)	2.051 (19)	2.9251 (17)	173.1 (16)
C1—H1B···O3	0.99	2.31	2.748 (2)	105
C5—H5···O4	0.95	2.27	2.8771 (19)	121
C7—H7···O2	0.95	2.40	2.7940 (18)	104
C11—H11B···O3^ii^	0.98	2.49	3.456 (2)	168
C12—H12A···O3	0.98	2.39	2.957 (2)	117
C13—H13C···O3	0.98	2.52	3.073 (2)	116

Symmetry codes: (i) *x*+1/2, *y*, −*z*+3/2; (ii) −*x*+3/2, *y*+1/2, *z*.
